# Myocardial Infarction in Young Individuals: A Review Article

**DOI:** 10.7759/cureus.37102

**Published:** 2023-04-04

**Authors:** Anupam Sood, Akhilesh Singh, Charuta Gadkari

**Affiliations:** 1 Department of Emergency Medicine, Jawarhalal Nehru Medical College, Datta Meghe Insititute of Higher Education and Research, Wardha, IND

**Keywords:** risk factors, coronary artery disease, smoking, myocardial infarction, young

## Abstract

Although myocardial infarction (MI) primarily affects patients over the age of 45, it can also affect young women and men. Still, when it occurs at an early age, it has severe morbidity and psychological and financial burdens for the patient and his or her relatives. Four classes can be used to categorize the causes of MI in individuals below the age of 45. These are drug abuse-related MI, hyper-coagulable conditions, atheromatous coronary artery disease (CAD), and non-atheromatous CAD. There is a significant overlap between each category. Elevated blood pressure, smoking, diabetes, obesity, high cholesterol, inactivity, an unbalanced diet, binge drinking alcohol, and related substances are all risk factors. The primary mechanism of an MI is typically the total obstruction of a vessel caused by breaking an atheromatous plaque. This article covers the research and focuses on the practical concerns related to young adults with MI.

## Introduction and background

The most significant cause of death for individuals in the West is coronary heart disease (CHD) [1-3]. The fatal symptom of CHD is myocardial infarction (MI), which might appear as a sudden demise. Although MI primarily affects individuals over the age of 45, it may also be seen in young men or women. Luckily, it rarely occurs in a population under the age of 45 [4]. When it occurs at a younger age, this illness has severe morbidity, psychological impacts, and financial burdens for the patient and his or her relatives. The protection provided to youth has gradually been destroyed by the rising prevalence of CHD risk factors (RF) in young adults, such as cigarette smoking, increased weight, and inactivity. The term MI describes the loss of cardiac muscle tissue (infarction) brought on by ischemia injury or the deprivation of oxygen to the myocardium. It is one type of acute coronary syndrome (ACS), which is described as an abrupt or brief shift in symptoms associated with blood flow in the heart. In contrast to unstable angina, the other type of ACS, MI, happens when cell death occurs, which can be confirmed by a blood test for biomarkers like cardiac troponin [5].

## Review

Methodology

**Figure 1:**
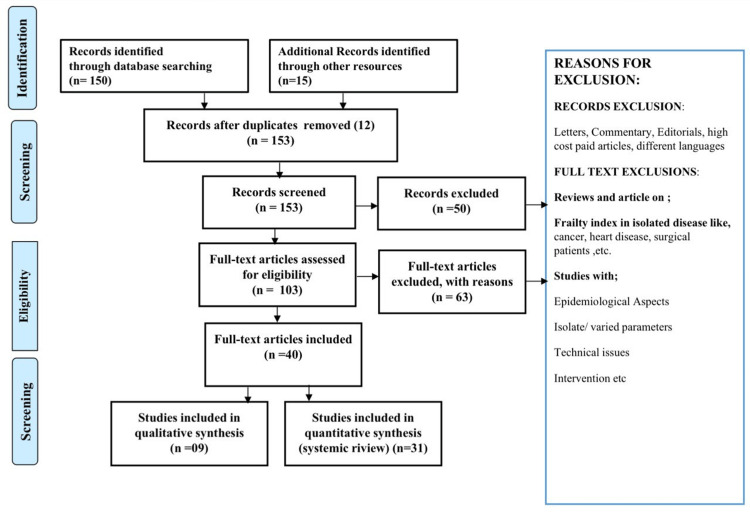
PRISMA methodology of the search strategy

We searched the Central and MEDLINE databases using the Cochrane Library and PubMed. The PubMed search technique was thus customized for each database: ("risk factors for coronary artery disease "[Title/Abstract] AND "young onset myocardial infarction"[Title/Abstract]) OR "Smoking and myocardial infarction"[Title/Abstract] OR " [Title/Abstract] OR "myocardial infarction"[Title/Abstract]. We also looked through the reference lists of potentially relevant papers to find additional studies. These electronic searches yielded studies, which were then analyzed alongside relevant sources in their bibliographies. Original studies written in English that evaluated RF, diagnosis, and treatment were included. The Preferred Reporting Items for Systematic Reviews and Meta-Analyses (PRISMA) research methodology is depicted in Figure 1.

Epidemiology

CHD is becoming less seen across all age groups in the United Kingdom. In reality, between 1992 and 2012, the condition was discovered to be present in 0.5% of males and 0.18% of women between the ages of 35 and 44, as well as 20.5% of men and 17.1% of women over the age of 60 [6]. In fact, due to atypical presentations and reluctance to submit themselves for further examinations, the number of younger patients may be lower than what they are [7]. However, it was discovered that just 3% of all CHD patients were in the younger group, defined as those under the age of 40 [8]. In the near future, this will lead to a rise in disease burden. Cigarette smoking is the leading RF for cardiac problems and has been proven to be more and more widespread in young individuals, where it can reach up to 8-10%. In the United Kingdom, it was discovered that girls who smoked more frequently and for a more extended period had a heavier smoking burden [9]. This would affect the protection of the heart provided by hormones like estrogen in young women. Increased weight is a significant concern in young people and children, and it has been elevated by three times in the United Kingdom in the past 20 years [10-12]. The use of cocaine is one of the frequent causes of manifestation of pain in the chest among younger individuals and can develop in MI [13,14]. It is almost transparent that the incidence of CHD, as predicted, has increased in patients aged less than 45.

Risk factors

**Figure 2:**
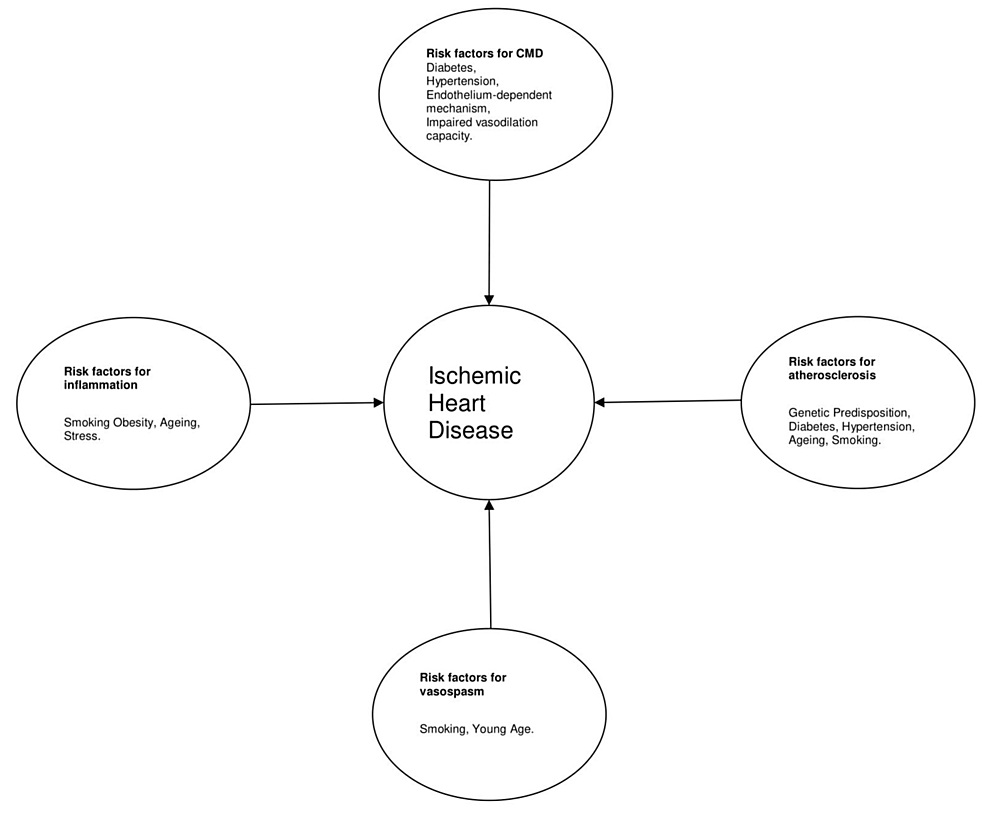
RF for the development of ischemic heart disease CMD: coronary microvascular dysfunction Source: Original

Cigarette smoking, cholesterol levels, diabetes, hypertension, increased weight, food habits, physical inactivity, and alcohol consumption were all considered RF. Psychological stress and the male gender are the major RF in younger individuals [15,16]. Post-COVID-19 infection and post-COVID-19 vaccination may also trigger MI in young people. More critical factors included smoking, lipid problems, hypertension, and diabetes. Cigarette smoking is the central and most common RF among younger individuals, while in the case of the elder population, hypertension, diabetes, and dyslipidemia are the most common RF [17,18]. Smoking is the only element that may be changed entirely. Smoking tobacco speeds up the onset of atherosclerosis by decreasing tissue oxygenation, harming the vascular endothelium, and increasing sympathetic nervous system activity. Smoking also increases platelet aggregatory activity, which aids in developing intravascular clots [19]. The most common RF in the 17 to 45 age category were smoking (approximately 57%), dyslipidemia (approximately 52%), and hypertension (50%), and approximately 91% of patients had at least one RF. One out of five patients had diabetes and obesity, and one out of every 10 people who had their first acute myocardial infarction (AMI) also used drugs. The most common conditions in people aged 45 to 59 were hypertension (60%), dyslipidemia (57.5%), and smoking (52%), and 92% of patients had at least one RF. At the time of the first AMI, diabetes mellitus was there in one in four instances, obesity in one out of six, and drug usage in one out of 20 cases. In both age groups, Hispanic patients have a higher rate of diabetes mellitus than other groups; Asian/Pacific/Islander patients had increased rates of dyslipidemia; Black patients had a higher frequency of elevated blood pressure (BP), increased weight, and substance misuse; and White patients had increased rates of smoking than other racial groups. The most common risk element during an initial AMI is dyslipidemia in individuals who were White (55%), Hispanic (50%), and Asian (approximately 56%) racial groups, while elevated BP was one of the most common RF in Blacks (about 64%). It is alarming that some RF are highly prevalent in the younger population, particularly elevated BP (about 50%) and dyslipidemia (about 51.8%). These figures are significantly higher than the current estimates of hypertension (12.8% in men and 9.4% in women) and dyslipidemia (approximately 13% in men and 8% in women) in the general adult population of the United States aged 20 to 44 [20]. The 18 to 44 years patient group had a rate of 22.6%, with a tendency to increase, which is significantly more than the 4% prevalence rate of diabetes mellitus in the United States. The risk of recurrent AMI or fatal CHD in the first five years after a first AMI in people over 45 is as high as 17-20%. The elevated risk of such occurrences is linked to the presence of specific changeable RF through lifestyle modification [21]. Figure 2 summarizes RF for the development of ischemic heart disease.

Pathogenesis

There are four categories in which the pathogenesis of MI in individuals below 45 can be described.

Atherosclerotic CHD

The atheromatous process begins in infancy. In a necropsy examination of 760 young adults who passed away for various reasons, 20% of men and 5% of women had advanced CHD. The pathogenic causes of young people's atherosclerosis were connected to the typical RF that is the same as in adults. Smoking was linked to atheromatous processes for young patients, according to reports shown to be prevalent up to 92%. Smoking was found to be more in patients under 40 years of age than in those who were beyond 60. Patients who had a MI while they were younger than 45 years old were shown to have higher rates of lipid abnormalities, particularly hypertriglyceridemia and low high-density lipoprotein. In addition to overt diabetes, a 65% prevalence of decreased glucose tolerance was identified in survivors of MI under 45 years old. In younger people, traditional RF is more important. In addition, the development of hyperhomocysteinemia and variable level of lipoprotein-A may have the same clinical effects [22,23].

Non-atheromatous Coronary Artery Abnormalities

Congenital coronary artery abnormalities are a possibility when MI strikes young adults. These are highly uncommon. Myocardial bridging (coronaries embedded within a tunnel in the myocardium) includes systolic contraction, which can result in substantial ischemia during contraction and can lead to MI. Surgical procedures and percutaneous intervention were both found to be medical management options more effective in this group of patients [22,23].

Recreational Drug Use

The use of cocaine is linked to many cardiac issues, including MI. Cocaine usage was associated with the clinical presentation in 48% of young patients who were referred to the emergency room with non-traumatic chest discomfort. A thorough background is essential since cocaine effects might manifest up to 72 hours after consumption. The majority of individuals who misuse cocaine are also chronic smokers, which increases their risk of MI. In addition to MI, cardio-myopathy, tachyarrhythmias, and endocarditis have all been linked to cocaine usage. MI can arise from using marijuana and amphetamines. Additionally, excessive alcohol consumption has been reported to be connected to a young person acquiring MI even though the mechanism is not evident [23,24].

Hyper-Coagulable State

Recurrent arterial and venous thrombosis is linked to the anti-phospholipid syndrome. Young adults in their 30s are frequently affected by it. It could be primary or secondary and connected with autoimmune conditions, such as systemic lupus erythematous. These patients often exhibit enhanced platelet adhesiveness and early atherosclerosis [23,24].

Clinical presentation

Ninety percent of males and females reported experiencing chest pain, such as pressure, tightness, or discomfort. Women had an increased rate of pain symptoms (61.9%) which was not related to the chest compared to males (54.8%), which included gastrointestinal distress (nausea and stomachache), elevated heart rate, and breathlessness [25]. Various signs and symptoms include chest pain that feels like pressure, aching, tightness, and squeezing pain, which radiates to the left arm, shoulder, or back, sweating, exhaustion, heartburning sensation or indigestion, dizziness, and shortness of breath. The adult male presents more frequently with chest discomfort and sweating [22], but there is a significant overlap between the symptoms of men and women. On the other hand, women tend only to experience non-chest pain discomfort, presenting symptoms such as pain in the back or neck or "nausea or vomiting," which was discovered to be poorly understood [26]. Similar to how it applies to younger people, older people also exhibit more unusual symptoms [27-29]. One reason women and the elderly experience higher fatality rates may be due to this lack of awareness of unusual symptoms. One study found that only 24% of young adults with documented coronary artery disease (CAD) had stable angina [30]. Sixty-nine percent of people under the age of 45 who had a MI denied having any chest pain prior to the MI. Most patients' symptoms were observed to last less than a week. A thorough history would provide crucial information for the differential diagnosis of chest discomfort. The initial focus of clinical inspection should be on hemodynamic stability. Signs of sympathetic hyperactivity, in the form of elevated heart rate, perspiration, and any indications of prior abuse of injectable drugs, are essential [31].

Diagnosis of MI

Within 10 minutes of admission, a doctor should order and interpret an ECG, which is a vital diagnostic tool [32]. In patients with significant ST elevation on electrocardiography or suspected new left bundle branch block (BBB), immediate coronary angiography should be performed. Risk stratification and an algorithm for risk-based diagnosis and treatment should be applied to all patients. ECG criteria were modified in such a way that it also takes into account the age of the patient and gender-specific differences about leads V2-V3. ST-segment elevations are significant when the J-point is elevated by less than 0.15 mV in women, less than 0.25 mV in men under 40, and less than 0.2 mV in males above 40. An increase of less than 0.1 mV is diagnostic in all other leads. Patients with left or right BBB, early repolarisation, persistent ST elevations from a remnant aneurysm, purely posterior MI, or poorly positioned leads may have trouble interpreting their ECG. Concordant ST elevations may be the strongest predictor of continued AMI in individuals with established left BBB. At the same time, more complicated algorithms do not seem to offer enough diagnostic assurance [33]. Cocaine consumption is linked to abnormal ECG, commonly seen as dynamic ST-segment elevation. Suppose the patient presents to the hospital emergency room as soon as possible after the commencement of chest pain. In that case, ST elevation on ECG may be detected. If precious time is lost, the pain in the chest and ECG abnormalities quickly disappear and may be missed. Vasodilators are given to individuals with coronary cocaine use-related arterial spasms [34]. After 12 hours of chest discomfort, patients have a high chance of abnormal Q waves. An arbitrary T-wave modification, ST wave inversion, and depression are observed in patients with coronary arteries that are partially blocked. With pleuritic pain and concave upward ST-segment elevation in lateral leads, myopericarditis may be present [35].

Cardiac Troponin: A Gold Standard Biomarker

Cardiac enzyme levels are consistently elevated. Troponin T elevation (cardiac specific) is the most precise indicator of cardiac injury. There are three subtypes of cardiac troponin, which regulate the myocardial contractile machinery (T, I, and C). Elevated levels signify myocardial damage because cardiac troponin T (cTnT) or troponin I (cTnI) expression occurs solely in cardiomyocytes [36]. A biphasic release kinetics causes an early peak to be seen within 24 hours, and the contraction apparatus proteolytic degradation causes a plateau after 48-72 hours [37,38]. While constant readings throughout serial measures indicate chronic myocardial injury, a distinct spike and decline in troponin levels or a considerably raised level of troponin during admission indicate AMI. A more likely occurrence of AMI is connected to a highly noticeable shift. If initial troponin readings are elevated, an earlier American National Academy for Clinical Biochemistry Guidance regarded a delta change of 20% or more as noteworthy. A rise or decline of 50% or more was suggested by a committee of the European Society of Cardiology (ESC). The 0-h/1-h rule-in and rule-out algorithms are defined by assay-specific absolute cut-off levels in the 2015 ESC guideline on managing non-ST-elevation ACS. High-sensitivity assays should only be described as having detectable troponin values in more than 50% of healthy people [39]. Patients may have a false-positive creatinine kinase rise with the abuse of cocaine.

Echocardiography

The diagnosis of non-ischemic causes of chest discomfort, such as myocarditis, valvular illness, cardiomyopathies, pulmonary embolism, or aortic dissection, is also aided by echocardiography. Echocardiography is also the preferred technique for identifying problems such as ventricular wall rupture or subsequent mitral valve regurgitation following papillary muscle rupture or ischemia [40].

MRI

Although less common and accessible than echocardiography, cardiac MRI is particularly useful in diagnosing myocardial illness.

Coronary Angiography

It gives essential information about the presence of any abnormalities in the coronary artery, and if present, it tells about the extent and identification of the offending lesion. New regional wall motion abnormality in echocardiography, myocardial scarring in MRI or nuclear testing, or an intra-coronary thrombus during coronary angiography, along with a significant spike or decline in cardiac troponin, are currently accepted diagnostic criteria for MI.

Management

Younger patients' first care for MI deviates slightly from typical adult management. All patients should be given first doses of oxygen, nitrates, diamorphine, and aspirin. Statins are also used, which have anti-inflammatory properties. For those patients that have a history of cocaine abuse, beta-blockers should be avoided in them because the chest ache paradoxically becomes worse. Benzodiazepines are recommended for the initial treatment of MI in cocaine abuse. These patients should continue receiving nitrates to prevent coronary spasms. Expert opinion on instability should be obtained among those with unstable hemodynamics, and coronary angiography and intervention should be considered. Thrombolytic treatment should be made available to patients with persistent ST elevation due to cocaine that has not improved with the help of nitrates. Younger individuals appear to tolerate thrombolytic drugs better. Risk stratification should be used after the initial management of patients with non-ST-segment elevation MI. Based on persistent or dynamic ECG abnormalities, a greater degree of cardiac increase enzymes, and extra RF such as diabetes mellitus, high-risk patients should be directed to experts so they can determine whether early coronary angiography and intervention are necessary or not. Many younger individuals have normal coronary arteries. Therefore, coronary angiography is not always administered to them. Exercise stress testing is a valuable tool for risk categorization in patients with existing MI. The majority of the young patients who completed stage three of the Bruce regimen (nine minutes or longer) were discovered to have no abnormalities in their coronary arteries. The majority of MI patients often undergo coronary angiography. As was previously noted, due to the greater likelihood of discovering a normal coronary artery, this may not be given as a standard option to every affected patient. The possibility of finding an aberrant coronary artery increases in people at high risk, such as diabetes mellitus, dyslipidemia, and a family history of early CHD. People with severe left ventricular dysfunction should be provided with coronary angiography since early revascularization in the form of percutaneous transluminal coronary angioplasty and coronary artery bypass graft surgery improves their prognosis [38-40].

## Conclusions

Fortunately, MI is rare in young individuals under the age of 45. However, it can still be a severe issue for the patient and the managing physician. It has a terrible impact on young people with sedentary lifestyles. A patient's age, various RF, clinical manifestations, and prognosis must all be considered. The increased frequency of CHD and various RF indicates the beginning of an alarming trend. In suspected MI patients who are less than 45 years old, abuse of substances, abnormalities in a coronary artery, premature CAD, and hypercoagulable status must be considered. After early stabilization, risk stratification should come next. Stratification and early revascularization should be provided since they will produce better clinical results. There is a lot of significance of secondary preventable measures for all newly hospitalized MI patients; if not prevented, the long-term mortality rate can be as high as one-third of the cases.
